# Gut microbiota, a hidden protagonist of traditional Chinese medicine for acute ischemic stroke

**DOI:** 10.3389/fphar.2023.1164150

**Published:** 2023-04-13

**Authors:** Lin Gao, Xiuwen Xia, Yinqi Shuai, Hong Zhang, Wei Jin, Xiaoyun Zhang, Yi Zhang

**Affiliations:** ^1^ Emergency Department, Chengdu University of Traditional Chinese Medicine Affiliated Hospital, Chengdu, Sichuan, China; ^3^ School of Basic Medical Sciences, Chengdu University of Traditional Chinese Medicine, Chengdu, Sichuan, China; ^2^ School of Clinical Medicine, Chengdu University of Traditional Chinese Medicine, Chengdu, Sichuan, China; ^4^ Geriatric Department, Chengdu University of Traditional Chinese Medicine Affiliated Hospital, Chengdu, Sichuan, China

**Keywords:** acute ischemic stroke (AIS), traditional Chinese medicine (TCM), gut microbiota, Chinese herbs, brain-gut-microbe axis (BGMAs), immunology and inflammation, intestinal barrier, microbial metabolites

## Abstract

Acute ischemic stroke (AIS) is one of the leading diseases causing death and disability worldwide, and treatment options remain very limited. Traditional Chinese Medicine (TCM) has been used for thousands of years to treat ischemic stroke and has been proven to have significant efficacy, but its mechanism of action is still unclear. As research related to the brain-gut-microbe axis progresses, there is increasing evidence that the gut microbiota plays an important role during AIS. The interaction between TCM and the gut microbiota has been suggested as a possible key link to the therapeutic effects of TCM. We have compiled and reviewed recent studies on the relationship between AIS, TCM, and gut microbiota, with the expectation of providing more ideas to elucidate the mechanism of action of TCM in the treatment of AIS.

## 1 Introduction

Stroke is an acute focal injury to the central nervous system (CNS) caused by vascular causes and is the leading cause of disability and death worldwide (Sacco et al., 2013), with acute ischemic stroke (AIS) accounting for 71% of all strokes (Campbell et al., 2019). Treatment options for AIS are still very limited. Many of the agents shown in preclinical studies to improve neuronal damage do not have significant real-world efficacy (O’Collins et al., 2006). Currently, intravenous thrombolysis and endovascular thrombectomy remain the treatment of choice for AIS, but many patients are unable to access these treatments in a timely manner due to the underlying disease and time of onset (Emberson et al., 2014; Saver et al., 2016). Ischemia/reperfusion (I/R) injury should not be overlooked, as it is commonly seen in patients following endovascular treatment (Shaik et al., 2021). TCM has a long history of treating AIS. Over thousands of years of practice and heritage, hundreds of herbal formulae have been created and applied in the treatment of stroke. Many Chinese herbs, such as Ligusticum chuanxiong, Astragalus membranaceus, Radix Salvia Miltiorrhizae, Carthamus tinctorius L., Angelica sinensis, etc., have been found to be effective in improving the prognosis of AIS (Zhao et al., 2022). Studies in pharmacology have found that many herbs have active effects, such as antioxidative stress, antiapoptotic, anti-inflammatory, proangiogenic, and neuroprotective effects (Zhu et al., 2021a). Therefore, herbal medicine has great potential for the treatment of AIS and deserves to be explored and studied.

**FIGURE 1:**
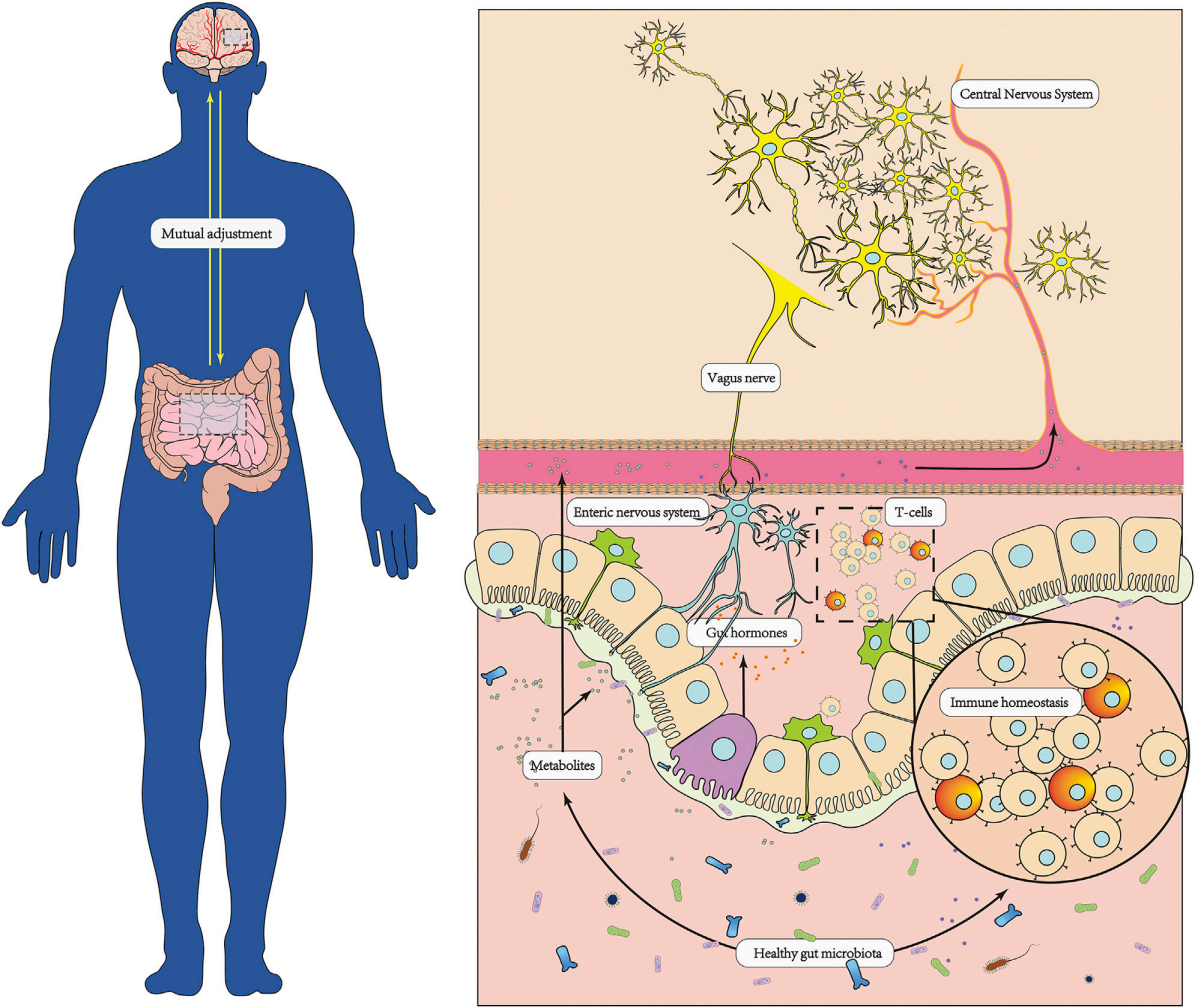
In healthy conditions, the brain and the gut microbiota have bidirectional regulation. The brain regulates the function of the gut through multiple neurological, endocrine and immune pathways, providing a stable and healthy environment for the colonization of the gut microbiota. Meanwhile, the gut microbiota plays an important role in facilitating the conversion and absorption of nutrients, maintaining homeostasis of the enteric and organic immune systems, protecting the integrity of the intestinal mucosal barrier, and promoting the development of the nervous system.

Recently, as the concept of the gut-brain-microbe axis (GBMAx) has become clearer (Figure 1), the importance of the gut microbiota and its metabolites in the course of AIS has been gradually revealed (Rhee et al., 2009; Pluta et al., 2021). In short, the disturbance of the gut environment caused by AIS can lead to a disorder of the gut microbiota and its metabolites, and this breakdown in microbiota diversity can in turn further aggravate AIS (Arya and Hu, 2018). The gut microbiota and its metabolites can influence inflammatory damage in the ischemic brain through direct regulation and indirect intervention (Yamashiro et al., 2017; Chen et al., 2019c; Durgan et al., 2019; Huang and Xia, 2021). Disorders of the gut microbiota can also damage the gut mucosal barrier, leading to the spread of harmful toxins and bacteria into the peripheral organs, causing infections and even sepsis (Crapser et al., 2016; Roubalová et al., 2020). Therefore, regulating the gut environment and maintaining the stability of the gut microbiota and metabolites is considered a new therapeutic direction for AIS. Studies have found that TCM plays crucial roles in promoting health and treating disease by interacting with the gut microbiota (Chen et al., 2016). Chinese herbs have a significant impact on the host gut environment in a variety of ways, including inhibiting pathogenic bacteria, promoting probiotics, regulating key flora metabolites, and maintaining the integrity of the intestinal barrier (Zhang et al., 2012; Zhao et al., 2012; Guo et al., 2015; Liu et al., 2018; Nie et al., 2019). Meanwhile, many herbal compounds effective in treating AIS have been found to require a transformation of the gut microbiota before they can exert their active effects (Gong et al., 2020). Increasing evidence indicates that the gut microbiota and its metabolites may be the key to the efficacy of TCM in the treatment of AIS. We have collated and reviewed recent studies on AIS, TCM, and gut microbiota for the purpose of providing more data on the mechanism of action of Chinese herbs in the treatment of AIS.

## 2 Chinese herbal medicine for AIS has advantages

Traditional Chinese herbs have the advantages of being multicentric, multitargeted, holistically regulated, and having few side effects in the treatment of AIS (Fan et al., 2020; Li et al., 2022b; Ye et al., 2022) Several meta-analyses and systematic evaluations of proprietary herbal compound preparations have shown significant effects on the degree of neurological deficits, the speed of neurological recovery, and the ability to perform activities of daily living in patients with AIS (Lyu et al., 2020a; 2020b; Liu et al., 2021; Tian et al., 2021; Wang et al., 2022c). Recent pharmacological studies have found that Chinese herbal medicine can treat AIS through several mechanisms, including protecting neurological function, promoting microvascular regeneration, and improving energy metabolism in ischemic cells.

Many herbs have been found to promote neuroprotection and reduce excitotoxicity, oxidative stress, apoptosis, inflammation and autophagy of neurons in ischemic conditions (Zhu et al., 2022). Gastrodin is the main active ingredient of the Chinese herb Gastrodia. Gastrodin can exert antioxidant and anti-inflammatory bioactivities in the treatment of cerebral ischemia and reduce neuroapoptosis in damaged brain tissue by inhibiting the Wnt/β-Catenin pathway (Qiu et al., 2019). Ginsenoside R1 is the most abundant active ingredient in ginseng, and many scholars believe that R1 may have beneficial therapeutic effects as a neuroprotective agent in AIS. Li et al. (2017) showed that ginsenoside Rg1 increased the survival rate of ischemic neural stem cells by reducing oxygen-glucose deprivation (OGD)-mediated oxidative stress and p38/JNK2 phosphorylation. Chu et al. (2019) revealed that R1 inhibits miR-144 activity and activates the Nrf2/ARE pathway to reduce oxidative stress injury in neuronal cells after ischemia‒reperfusion. Zheng et al. (2019) found that R1 attenuates ubiquitinated protein aggregation in ischemia-reperfused brain tissue and reduces the activation of NF-κB and IκBα for neuroprotective effects. Baicalin (BA), the main active component of Scutellaria baicalensis, has been found to have neuroprosthetic effects by stimulating the conversion of astrocytes into reactive astrocytes and secreting more brain-derived neurotrophic factor (BDNF), thereby promoting dendritic growth and branching of nerve cells (Li et al., 2021). Salidroside (Sal) is an active component of Rhodiola rosea L. with anti-inflammatory, antioxidant and anti-apoptotic effects, and recent studies have found that Sal promotes dendritic and synaptic plasticity through the FGF2-mediated cAMP/PKA/CREB pathway (Li et al., 2020b).

Promoting capillary angiogenesis is considered to be an effective way to combat ischemic diseases, and many herbal formulations, with monomeric compounds that have a pro-angiogenic effect, have an ameliorative effect on improving persistent ischemia in brain tissue in acute cerebral infarction (Bu et al., 2020). For example, the famous Chinese herbal formula BUYANGHUANGWU Tang for stroke was found to increase capillary density in brain tissue to improve blood supply to the brain in a rat model of focal cerebral ischemia/reperfusion injury (CI/RI) (Shen et al., 2014; Yang et al., 2015). Angelica Astragali Radix (AR), one of the constituent drugs of Tonic Yang Returning Five Soup, was found to promote angiogenesis in cerebral ischemic rats by activating the p38 MAPK/HIF1α/VEGF-A/vWF signaling pathway in aqueous extract of Angelica (Cheng et al., 2017). Panaxatriol saponins (PTS), an extract of Panax notoginseng, were found to enhance angiogenesis in the ischemic border zone by activating the Shh signaling pathway to upregulate vascular endothelial growth factor (VEGF) and angiopoietin-1 (Ang-1) expression (Hui et al., 2017). Bilobalide B and ginkgolide K were found to activate the Akt/eNOS pathway and JAK2/STAT3 pathway, respectively, to promote angiogenesis in ischemic brain tissue (Chen et al., 2018; Zheng et al., 2018).

Improved intracellular energy metabolism can effectively protect ischemic semidark zone nerve cells. Dan Hong injection (DHI) is a compound Chinese medicine used for the treatment of acute ischemic stroke. Dan Hong injection can improve energy metabolism in ischemic brain cells and reduce nerve damage in the semidark zone by restoring mitochondrial activity and cytoplasmic glycolytic activity (Zeng et al., 2021; Du et al., 2023). Herba leonuri (HL) is an herbal medicine with blood-activating properties and is often used to treat AIS. Leonurine, an alkaloid of HL, was found to reduce mitochondrial swelling in ischemic brain tissue and restore mitochondrial function to some extent (Qi et al., 2010).

## 3 Brain-gut-microbiota interactions in AIS

**FIGURE 2:**
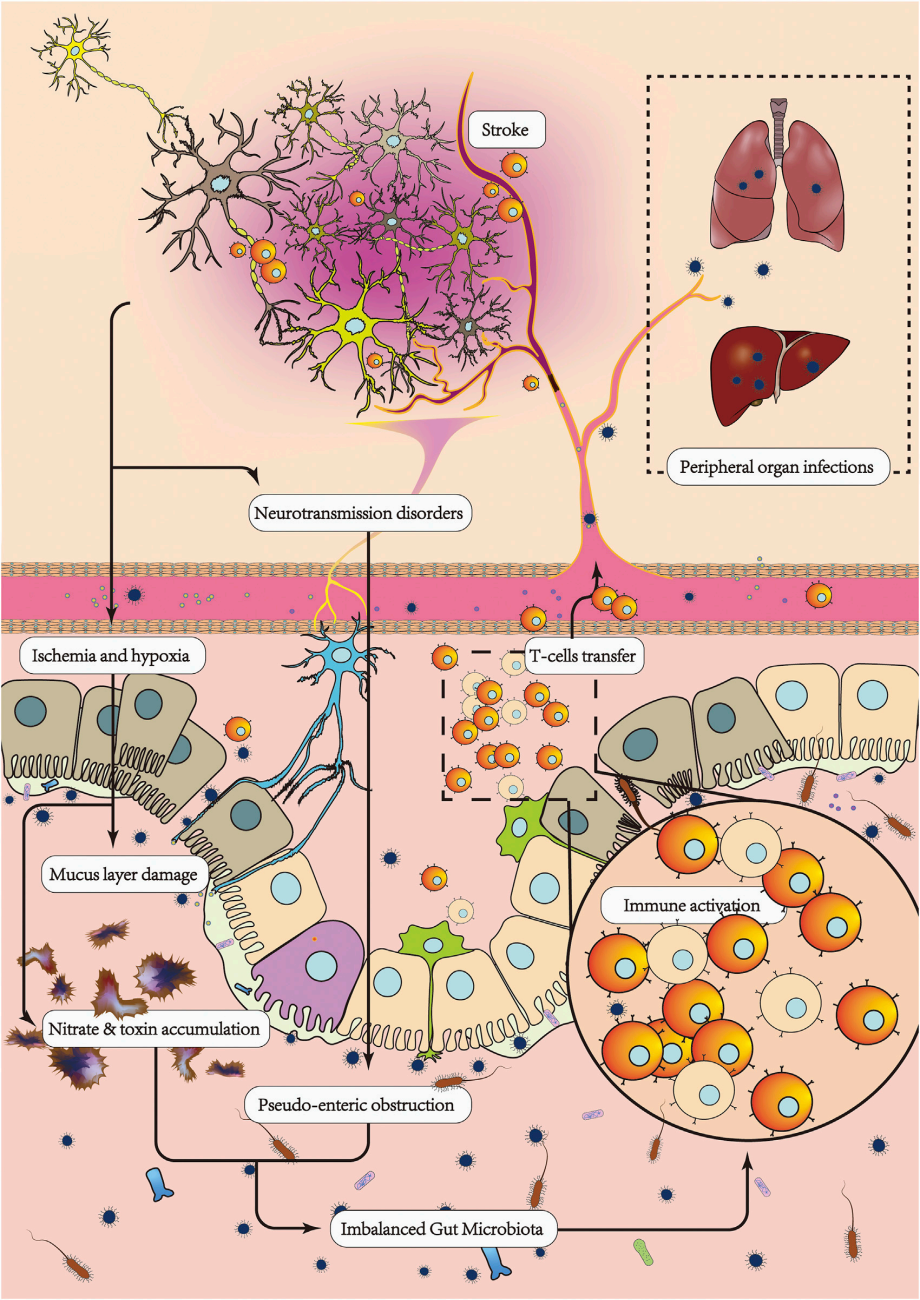
After AIS, the brain-gut regulatory mechanism is inhibited, resulting in an imbalance in the intestinal environment, leading to a decrease in probiotic bacteria and an increase in pathogenic bacteria. Enteric nerve conduction disorders cause pseudointestinal obstruction and constipation, which leads to a failure of pathogenic bacteria and harmful substances in the intestinal tract to be excreted in the feces in a timely manner. On the other hand, a disturbed gut microbiota has a serious impact on AIS. Intestinal immune homeostasis is disrupted, the proinflammatory/anti-inflammatory lymphocyte ratio is imbalanced, and more proinflammatory lymphocytes are transferred from the gut to the damaged brain, leading to increased inflammatory damage and apoptosis in the brain. Massive loss of probiotic bacteria and thinning of the intestinal mucus layer leads to increased intestinal barrier permeability, making it easier for pathogenic bacteria to cross the intestinal wall and enter the circulation, causing peripheral organ infections and sepsis. Dysregulated secretion of metabolites from the gut microbiota, such as decreased SCFAs, increased TMAO, increased LPS, increased excitatory neurotransmitters, and decreased inhibitory neurotransmitters, leads to a poorer prognosis for AIS.

It has been found that the gut microbiota plays an important role in facilitating food conversion and absorption, regulating body metabolism, maintaining homeostasis of the immune system, protecting the integrity of the intestinal mucosal barrier, and preventing pathogenic invasion (Clemente et al., 2012; Bunker et al., 2015; Hamilton et al., 2015; Gomaa, 2020). The gut microbiota and its fermentation/degradation products have been directly or indirectly linked to the development of many diseases (Han et al., 2010; Smith, 2015; Nie et al., 2019). Growing evidence indicates a subtle interaction between the gut flora and the injured brain after AIS (Figure 2). Brain damage can lead to gut dysfunction and microbiota disruption (Camilleri, 2021), and the gut microbiota influences the severity of neurological damage by modulating the host’s immune metabolism in reverse (Pluta et al., 2021).

### 3.1 AIS leads to disorder of the gut microbiota

Gut microbes are so numerous and able to coevolve with their hosts that their gene count far exceeds that of the host (Sekirov et al., 2010; Loscalzo, 2013). In addition to conventional factors such as diet, age and sex, the gut microbiota is also regulated by the nervous system. Many studies have demonstrated that the brain can regulate gut function and the composition of the gut microbiota through multiple neural, endocrine and immune pathways (Mazmanian et al., 2005; Barugh et al., 2014; Houlden et al., 2016). Therefore, when the brain is injured by AIS, the gut microbiota also suffers a serious impact. It was found that the gut microbiota of stroke patients is clearly different from that of healthy volunteers, with more opportunistic pathogenic bacteria such as *Parabacteroides, Oscillospira, Enterobacteriaceae, Megasphaera, Oscillibacter* and *Desulfovibrio* present in the gut of stroke patients compared to healthy individuals whose gut is rich in *Prevotella, Roseburia, fecalibacterium*, etc., (Yin et al., 2015; Xia et al., 2019). The disruption of the gut microbiota due to AIS may be related to intestinal wall damage and abnormal intestinal function. AIS can cause ischemia and hypoxia in the intestinal wall, leading to a large accumulation of nitrates in the gut, which allows for a decrease in the abundance of beneficial bacteria and an overgrowth of pathogenic bacteria (Xu et al., 2021b). Moreover, abnormalities in intestinal nerve conduction caused by AIS can lead to intestinal disorders such as pseudoenteric obstruction and constipation, which prevent the accumulation of pathogenic bacteria and harmful substances in the intestinal tract from being excreted in time with the feces (Camilleri, 2021). This top-to-bottom (brain-gut-microbiota) disruption was found to persist for up to 12 months after the onset of AIS (Chen et al., 2019c).

### 3.2 Gut microbiota affects the prognosis of AIS

Disorders of the gut microbiota have a significant impact on the prognosis of AIS. The accumulation of pathogenic bacteria in the gut can cause an imbalance in intestinal immunity and increased differentiation of pro-inflammatory lymphocytes, with the result that more pro-inflammatory lymphocytes are transferred to the brain and increase inflammatory damage to brain tissue and apoptosis of nerve cells. Without the protection of probiotics, the intestinal barrier becomes weaker, and pathogenic bacteria have a higher chance of crossing the intestinal barrier into the internal environment and causing infections and sepsis. Metabolic imbalances caused by disorders of the gut microbiota are also not to be overlooked, and gut flora metabolism/degradation products can affect the extent of brain damage in several ways.

#### 3.2.1 Increased brain transfer of intestinal proinflammatory T lymphocytes

Studies have found that disorders of the gut microbiota can lead to more proinflammatory T lymphocytes being transferred from the gut to the brain after AIS and exacerbate brain tissue inflammation. Inflammation is the main cause of ongoing brain damage after AIS (Tuttolomondo et al., 2009; Jin et al., 2010; Sorby-Adams et al., 2017), and the transfer of intestinal T lymphocytes to the brain is thought to be a key link in the induction of inflammation in brain tissue (Gelderblom et al., 2009; Liesz et al., 2009; Shichita et al., 2009; Kleinschnitz et al., 2010; Meng et al., 2019; Brea et al., 2021). The gut microbiota is a powerful regulator of the intestinal immune environment, and different species of flora can regulate the differentiation of a variety of T lymphocytes through direct or indirect contact with intestinal dendritic cells (Mazmanian et al., 2005). TH1, TH17 and γδ T cells can exacerbate inflammatory brain injury through the release of proinflammatory factors such as IL-17, TNF-α, γ interferon, perforin, and neutrophil aggregation factor cxcl1 (Shichita et al., 2009; Liesz et al., 2011; Gelderblom et al., 2012). In contrast, Treg cells can attenuate brain injury by suppressing immune responses, which may be associated with the release of IL-10 and inhibition of the differentiation of proinflammatory lymphocytes such as intestinal γδT cells (Liesz et al., 2015; Benakis et al., 2016). Disruption of intestinal immune homeostasis due to disorders of the gut microbiota and upregulation of pathogenic bacteria abundance leads to increased differentiation of pro-inflammatory T lymphocytes and decreased numbers of Treg cells (Benakis et al., 2016), which leads to more pro-inflammatory T lymphocytes transferring to injured brain tissue and exacerbating inflammatory damage (Feng et al., 2019b). Therefore, maintaining a stable gut microbiota is important in reducing inflammatory damage to brain tissue after AIS.

#### 3.2.2 Damage to the intestinal barrier and dissemination of pathogenic bacteria

Disorders of the gut microbiota can exacerbate the damage to the intestinal barrier caused by AIS, making it easier for pathogenic bacteria that have accumulated in the gut to cross the intestinal barrier and spread to peripheral organs, leading to infection and sepsis, which is an important cause of increased mortality from AIS (Crapser et al., 2016). A clinical sentinel study found that disorders of the gut microbiota in stroke patients were an independent risk factor for the development of pneumonia (Xia et al., 2021). Studies on stroke animals have also demonstrated that the bacteria causing pneumonia in MACO mice do indeed originate from the gut (Stanley et al., 2016). The integrity of the intestinal barrier function depends on the tight attachment of mucus proteins to the intestinal epithelium (Maria-Ferreira et al., 2018). It has been found that many probiotics are guarantors of the integrity of the intestinal barrier, such as A. muciniphila, a commensal bacterium of the intestinal mucus layer that degrades mucin for energy and forms a protective intestinal barrier (Paone and Cani, 2020), and *Lactobacillus*, which can prevent pathogenic bacteria from invading the intestinal wall by adhering to and colonizing the intestinal mucosa to increase intestinal epithelial tight junctions (Liu et al., 2011). Disorders of the gut microbiota result in the loss of probiotic bacteria, reduced secretion of intestinal tight junction proteins, decreased mucus levels, and increased intestinal permeability, which makes it easier for pathogenic bacteria to cross the barrier and disseminate to peripheral vital organs (Zhang et al., 2021). Therefore, maintaining a stable gut microbiota after AIS is important to protect the integrity of the intestinal barrier and reduce the incidence of poststroke infections.

#### 3.2.3 Metabolites of the gut microbiota influence the prognosis of AIS

Abnormal metabolite expression due to disorders of the gut microbiota has a significant impact on the prognosis of AIS. Short-chain fatty acids (SCFAs) are organic fatty acids produced by the breakdown of leftover nutrients in the gut microbiota and are considered to be key signaling molecules in the communication between the gut and the brain (Smith and Macfarlane, 1997; Garcia et al., 2008; Kotani et al., 2009; O’Mahony et al., 2015; Yissachar et al., 2017; Jameson and Hsiao, 2018). Lower levels of SCFAs in the feces of AIS patients compared to healthy groups are thought to be related to the loss of SCFA-producing bacteria in the gut (Li et al., 2019; Tan et al., 2021). SCFAs can promote neurological repair by modulating peripheral lymphocytes to influence brain microglial phenotypic differentiation (Sadler et al., 2020). A study found that reduced intestinal acetic acid may lead to a higher risk of 90-day malfunction in AIS patients (Tan et al., 2021). Preclinical studies have found that butyrate supplementation could improve the degree of brain edema and reduce the area of brain damage in MCAO rats (Chen et al., 2019b), which may be related to butyrate activating PI3K/Akt via GPR41/Gbc and attenuating neuronal apoptosis (Zhou et al., 2021). Trimethylamine N-oxide (TMAO) has been thought to be a gut microbial metabolite closely related to AIS (Méjean et al., 1994; Craciun and Balskus, 2012; Koeth et al., 2014; Fu et al., 2020). TMAO could be used as a biological risk predictor for stroke (Koeth et al., 2013; Tang et al., 2013; Zhu et al., 2016; Xu et al., 2021a). Meanwhile, elevated TMAO levels often indicate poor clinical outcomes in patients with AIS (Zhai et al., 2019; Tan et al., 2020). Elevated serum TMAO concentrations in MCAO rats lead to enlarged cerebral infarcts and increased neurological deficits (Zhu et al., 2021b). TMAO mediates the activation of multiple inflammatory signaling pathways, such as nuclear factor-Kappa B (NF-κβ), NOD-, LRR- and pyrin domain-containing protein 3 (NLRP3) inflammasomes, and MAPK/JNK in the brain and periphery, which may aggravate AIS (Praveenraj et al., 2022). Gut microbiota can remotely influence central nervous function by synthesizing neurotransmitters (Benakis et al., 2020). After AIS, gut microbiota disorder can lead to abnormalities in gut neurotransmitter metabolism, as evidenced by upregulated expression of excitatory neurotransmitters (e.g., Glu, ASP) and decreased expression of inhibitory neurotransmitters (e.g., GABA), resulting in neuro excitotoxic injury, and this brain injury would be reduced by correcting the neurotransmitter disorder (Gao et al., 2013; Bhuvanendran et al., 2019; Guo et al., 2021b). Lipopolysaccharide (LPS) is an immunogenic endotoxin from the microbiota that can promote neuroinflammation by direct mechanisms and/or by inducing the migration of peripheral immune cells to the brain (Lukiw et al., 2018). Polysaccharide A (PSA) is one of the metabolites of *Bacteroides fragilis* and has immunosuppressive effects on inflammation (Dasgupta et al., 2014; Wang et al., 2014; Johnson et al., 2015). Therefore, influencing metabolite expression by modulating the gut microbiota may be an effective way to improve AIS prognosis.

## 4 TCM treats AIS by interacting with the gut microbiota

**FIGURE 3:**
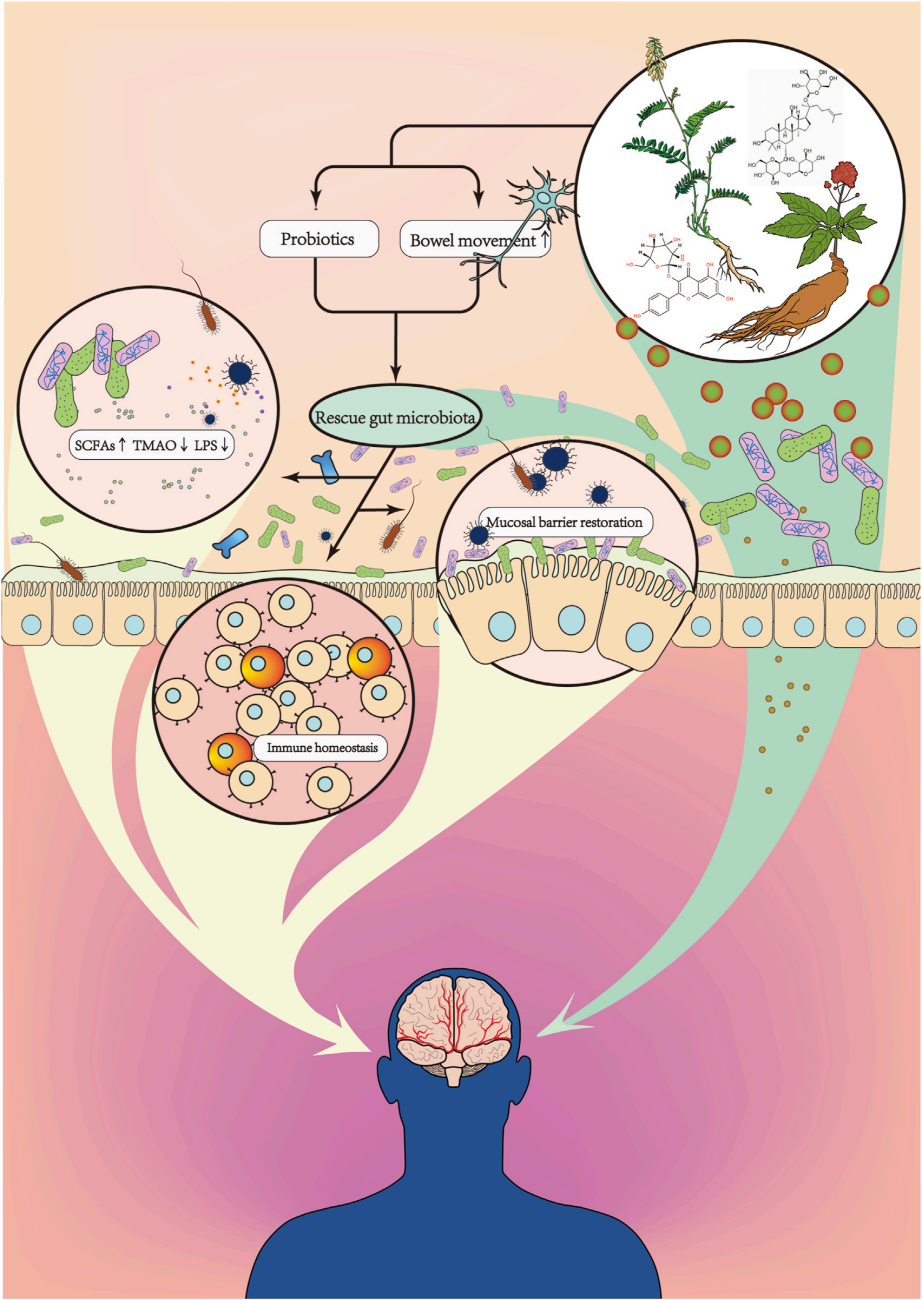
The interaction of traditional Chinese medicine with gut microbiota may be one of the mechanisms responsible for its efficacy against AIS. Chinese herbal medicines (including formulae and compounds) can restore gut microbiota diversity to varying degrees, bringing the intestinal flora closer to a healthy population after AIS. At the same time, traditional Chinese medicine can upregulate certain enteric neurotransmitters, increase intestinal motility, and promote the excretion of harmful substances. By correcting gut microbiota disorder, TCM influences intestinal microbial metabolite levels, improves the intestine’s inflammatory environment, attenuates neurohypoptosis, protects the integrity of the intestinal barrier, and reduces the escape of harmful bacteria. Meanwhile, many herbal compounds can be transformed by the action of the gut microbiota into structures with higher activity and bioavailability, which can then be absorbed by the intestine to exert therapeutic effects.

In recent years, there has been increasing evidence that the gut microbiota may play a key role in the treatment of AIS with TCM (Figure 3) (Feng et al., 2019a; Lin et al., 2021; Song et al., 2021; Tang et al., 2022). AIS can cause disruption of the gut microbiota, which is an important cause of poor prognosis in AIS, and Traditional Chinese herbs can correct this microbiota disorder. It was found that the use of Chinese herbal preparations (both formulae and compounds) could restore the diversity of the gut microbiota to varying degrees, increase the abundance of probiotic bacteria, reduce the abundance of conditionally pathogenic bacteria, and bring the gut microbiota closer to that of healthy populations after AIS(Cai et al., 2019; Zhang et al., 2020; Fu et al., 2021; Lin et al., 2021; Wang et al., 2022b; Cheng et al., 2022; Xu et al., 2022). To investigate how TCM regulates gut microbiota and how it exerts therapeutic effects, we searched for recently published studies on the regulation of gut microbiota by TCM in the treatment of AIS, including Chinese herbal formulae (Table 1) and herbal compounds (Table 2). In this way, we hope to elucidate the mechanism of action of TCM in treating AIS by modulating the gut microbiota and to provide scientific data for further research.

**TABLE 1:** Studies on the regulation of gut microbiota by Chinese herbal formulations.

Name	Formulary composition	Subject	Efficacy	Gut microbiota	Metabolites	Intestinal barrier	Citation
Xinglou Chengqi Decoction (XCD)	Dahuang (Rhei Radix et Rhizoma), Dan nanxing (Rhizoma Arisaematis Cum Bile), Gualou (Fructus et Semen Trichosanthis), Mangxiao (Nalrii Sulfas)	*Homo sapiens*	Improved NIHSS score and shortened spontaneous defecation time in AIS patients	_	_	_	Chen et al. (2020)
		C57BL/6 mice	Improves neurological function, alleviates cerebral infarction, and reduces apoptosis of neurons	Regulated SCFAs-producing bacteria like Verrucomicrobia and Akkermansia, and inflammation-regulated bacteria like Paraprevotella, Roseburia, Streptophyta, and Enterococcu	significantly increased the levels of short-chain fatty acids (SCFAs), especially butyric acid	_	Gao et al. (2021)
Tanhuo decoction (THD)	Huanglian (Coptidis Rhizoma), Dahuang (Rhei Radix et Rhizoma), Lianqiao (Forsythiae Fructus), Longdan (Gentianae Radix et Rhizoma), and Dan nanxing (Rhizoma Arisaematis Cum Bile)	*Homo sapiens*	Alleviated neurological impairment and phlegm-heat syndrome (including bad breath, yellow tongue coating, red tongue, sticky sputum, thirst for cold drinks, dry stool, and yellow urine.)	Increased Coprococcus, Dorea, Blautia, *Streptococcus*, Bifidobacterium, and *Lactobacillus*. Decreased *Clostridium*, Parabacteroides, and Phascolarctobacterium	Increased butyrate reduced propionate	_	Guo et al. (2022)
					Reduced the levels of LPS and TMAO		
		*Homo sapiens*		Increased Anaerostipes, Gemmiger, and Coprococcus, Bifidobacterium, Blautia, Ruminococcus, and *Streptococcus*	-	-	Guo et al. (2021a)
				Decreased: *Bacteroides*, Oscillospira, Eubacterium, Lachnospira, Odoribacter, Phascolarctobacterium			
NaoMaiTong	Renshen (Ginseng Radix et Rhizoma), Dahuang (Rhei Radix et Rhizoma), Gegen (Puerariae Lobatae Radix), Chuanxiong (Chuanxiong Rhizoma)	SD rats	Reduced cerebral infarction, improved neurological function	Increased Bacteroidaceae, Muribaculaceae, Ruminococcaceae, Lachnospiraceae, Turicibacter, and Bifidobacterium	restored 39 gut-derived metabolites	Reduced inflammation and damage of Intestinal Barrier, intestinal mucous layer was thickened, and the number of villi had recovered, the expressions of ZO-1 and MMP9 were observably recovered	Lin et al. (2021)
				Decreased: *Escherichia* Shaigella			
Qishiwei Zhenzhu Pills	such as myrobalan, pearl, agate, opal, bezoar, coral, musk, gold, silver, and Zuotai.ect	SD rats	effectively inhibit IL-6, IL-1β, and other inflammatory factors to effectively reduce the inflammatory response	Increased Firmicutes, L. johnsonii		Increased the villus height, villus width, crypt depth of ileum, and the mucosal thickness of the cecum and colon	Fu et al. (2021)
				Reduced Proteobacteria, *Escherichia Shigella*, L. reuteri			
PLR and CXR	Gegen (Puerariae Lobatae Radix) Chuanxiong (Chuanxiong Rhizoma)	SD rats	Improved neurological function and reduced complications of ischemic stroke	Increased Alloprevotella, Ruminococcaceae_UCG_005, Ruminococcaceae_NK4A214_group, Ruminococcaceae_UCG_004, Oscillospira, Lachnospiraceae_NK4B4_group, Akkermansia, and Megasphaera	Increased butyrate	Reduced the high intestinal permeability, enhanced the claudin-5 and ZO-1 levels, and declined the serum levels of DAO, LPS, and D-lactate significantly	Chen et al. (2019a)
Buyang Huanwu Decoction	Huangqi (Astragali Radix), Chishao (Paeoniae Radix Rubra), Chuanxiong Rhizoma), Danggui (Angelicae Sinensis Radix), Dilong (Pheretima), Taoren (Persicae Semen), Honghua (Carthami Flos)	SD rats	Improve neurological severity scores and alleviate neuronal damage; reduce the level of peripheral proinflammatory cytokines and inhibit neuroinflammation; regulated hippocampal metabolism	Increased *Lactobacillus*, Faecalibacterium, Ruminococcaceae_UCG-002, etc., Decreased Escherichia-Shigella, *Klebsiella*, *Streptococcus*, Coprococcus_2, *Enterococcus*, etc.	_	_	Tang et al. (2022)
HQ-HH	Huangqi (Astragali Radix), Honghua (Carthami Flos)	SD rats	Significantly reduced the neurological deficit scores, reduced the cerebral infarct volume, reduced the rate of necrotic neurons	Increased Blautia, Lachnospiraceae, Oscillibacter, and Bifidobacterium. Decreased Ruminococcaceae, *Bacteroides*, Phascolarctobacterium, and Desulfovibrionaceae	_	Increase the protein levels of ZO-1, occludin, and claudin-1, increased the protein levels of FXR, decreased fore bile acids including allolithocholic acid (AlloLCA), isolithocholic acid (IsoLCA), 3b-ursodeoxycholic acid (Beta-UDCA), and cholic acid (CA), decreased IL-17A expression and increased Foxp3 expression	Wang K. et al. (2022)
Tong-Qiao-Huo-Xue Decoction	Shexiang (Moschus), Taoren (Persicae Semen), Honghua (Carthami Flos), Dazao (Jujubae Fructus), scallion (Allium fistulosum L.), Shengjiang (Zingiberis Rhizoma Recens), Chuanxiong (Chuanxiong Rhizoma), Chishao (Paeoniae Radix Rubra), Yellow rice or millet wine	SD rats	Reducing stroke brain damage and neurological scores	Increased Allobaculum and Bifidobacterium	_	Improves the destruction of the small intestinal villi, ZO-1 upregulated and Claudin-2 downregulated, reduced plasma D -lactate, LPS and DAO. Significantly increased the number of Treg cells and upregulated IL-10 expression, reduced γδ T-cell numbers and decreased IL-17 expression	Zhang et al. (2020)
				Decreased Bacteroidetes			
Sanhuang Xiexin Decoction	Huanglian (Coptidis Rhizoma), Dahuang (Rhei Radix et Rhizoma), Huangqin (Scutellariae Radix), Danzhuye (Lophatheri Herba), Dannanxing (Rhizoma Arisaematis Cum Bile), Lianqiao (Forsythiae Fructus)	*Homo sapiens*	Reduction in the Fire and Heat Syndrome score of AIS and reduction in the incidence of ischemic cerebrovascular events at 3 and 6 months after endovascular intervention	_	Reduced plasma TMAO levels after endovascular intervention	_	Song et al. (2019)

**TABLE 2:** Studies on the regulation of gut microbiota by Chinese medicinal compounds.

Herb name	Medicinal compound	Action on the gut microbiota	Transformation through the gut microbiota	Citation
Dahuang/Rhei Radix et Rhizoma/Rhubarb Tangute Rhubarb		Increased abundance of *Bacteroides*, Alistipes, and Phascolarctobacterium, Reduced abundance of lachnospiraceae and romboutsia, and reduced serum TMAO and trimethylamine (TMA) levels (Enema)		Ji et al. (2021)
	Anthraquinone glycosides	Influencing intestinal neurotransmitter secretion through regulation of intestinal flora, increased the content of 5-HT, 5-HIAA, GABA, and decreased the content of Asp and Glu		Guo Y. et al. (2021)
			Be metabolized by intestinal flora to more readily absorbed anthraquinone aglycones	Li Q. et al. (2020)
Huanglian/Coptidis Rhizoma/Chinese Goldthread Rhizome				
	Berberine	Enriched the abundance of Roseburia, Blautia, Allobaculum, Alistipes, and Turicibacter, and changed the abundance of Bilophila, these flora are thought to increase the levels of intestinal SCFAs (acetic acid, butyric acid) and decrease the levels of TMAO.		Wu M. et al. (2020)
		Reduces the relative abundance of Desulfovibrio, Eubacterium and *Bacteroides*. and improves the Treg/Th17 balance in the gut		Cui et al. (2018)
		Berberine promotes the growth of A.muciniphila by stimulating mucus secretion in the colon and forms a dense protective intestinal barrier		Dong et al. (2021)
			Intestinal flora can improve the bioavailability of BBR by cut down it into the DhBBR form, a molecular form more readily absorbed by the intestine	Wang H. et al. (2022)
Renshen/Ginseng Radix et Rhizoma/Ginseng Root		Oral administration of Korean Red Ginseng KRG restores intestinal microbial diversity and increases the level of *Lactobacillus*		Lee et al. (2022)
		Fermented ginseng alleviated LPS-induced inflammation and increased intestinal barrier function in mice via the TLR4/MAPK signaling pathway		Fan et al. (2021)
	ginsenoside	R1 can improve inflammation in the gut by modulating intestinal flora, such as increasing the abundance of norank_f_Muribaculaceae, *Lactobacillus*, Allobaculum, and Akkermansia, and decreasing the abundance of Odoribacter, Clostridia_UCG-014, *Bacteroides*, and Turicibacter		Cheng et al. (2022)
			Ginsenosides Rb1, Rb2 and Rc can be converted by the gut microbiota into compound K with higher biological activity	Qi et al. (2011)
Gegen/Puerariae Lobatae Radix/Lobed Kudzuvine Root			Pueraria lobata fermented with Bifidobacterium breve increases the abundance of some anti-inflammatory flora in the intestine, such as Lactococcus and Ruminococcus	Choi et al. (2020)
	Puerarin	Increase the abundance of beneficial flora, including Lactobacillaceae and Bacteroidetes, and decrease the abundance of Ruminococcaceae, Prevotellaceae, and Burkholderiaceae		Xu et al. (2022)
		Puerarin reduces pathological changes such as thinning, shortening and uneven distribution of intestinal microvilli, and reduces endotoxin leakage from the intestine		Peng et al. (2013)
		Increased abundance of the Akkermansia muciniphila, a mucin-degrading bacterium, and increased expression levels of ZO-1, occludin, Muc2 and Reg3g in the intestine		Wang et al. (2019)
		Reduced the abundance of Ruminococcus 1 and RuminococcaceaeUCG-009 within Clostridia class to normal levels in lumen, and increased the production of acetate, propionate and butyrate		Wu Y. et al. (2020)
Huangqi/Astragali Radix/Mongolian Milkvetch Root Membranous Milkvetch Root		increased *Acetobacter* and *Escherichia*, and two metabolites, 7-keto-3A ·12-α-hydroxyalkanoic acid and deoxycholic acid		Liu et al. (2022)
	Astragaloside IV	Increased Anaerobacter, Romboutsia, Alkalibacteria, Canadidatus stoquefichus, Oligobacterium, Brautella, and Erysipelatoclostridum, decreased *Bacteroides*, Oscillibacter, Parabacteroides, Roseburia and Muribaculum.and increased butyric acid content		Gong et al. (2021)
		Increased abundance of Bifidobacterium, Blautia, Escherichia-Shigella, decreased abundance of Megamonas, Holdemanella, and *Clostridium*, and downregulated expression of the autophagic markers Beclin-1, Atg12, and LC3 II.		
Chishao/Paeoniae Radix Rubra/Red Paeoniae Trichocarpae		Increases the relative abundance of beneficial intestinal bacteria. Increases expression of ZO-1, occludin and Claudin-1 and inhibits the IL-23/IL-17 axis to regulate inflammation		Yan et al. (2022)
Danggui/Angelicae Sinensis Radix/Chinese Angelica	Angelica sinensis polysaccharide	regulated norank_f__norank_o__Clostridia_UCG-014, *Lactobacillus*, norank_f__Oscillospiraceae and norank_f__Desulfovibrionaceae, and upregulated Cldn5 to restore intestinal dysfunction such as tight junction disorder		
Danshen/Salviae Miltiorrhizae Radix et Rhizoma/Dan - shen Root		The ethanol extract of SMR significantly regulated Shuttleworthia, peptostreptococcaceae and *Pseudomonas*, while the water extract significantly affected *Peptococcus*, peptostreptococcaceae and Ruminococcus		Cai et al. (2019)
	tanshinones		Metabolic transformations such as methylation, demethylation, dehydrogenation, hydrogenation and hydroxylation take place with the involvement of the intestinal flora	Cai et al. (2019)
	salvianolic acids		Metabolic transformations such as glucuronidation, methylation and hydrogenation as tannic acid occur in the presence of intestinal flora	Cai et al. (2019)
	Salviae miltiorrhizae Radix et Rhizoma polysaccharide	Regulated abundance of Ruminococcus_gnavus, Desulfovibrio_C21_c20,Bifidobacterium_pseudolongum and *Clostridium*_cocleatum. Increased anti-inflammatory factors (IL2, IL10 and TGF-β) and decreased pro-inflammatory factors (IL-6 and IL23) in the intestine. Protects the function of the intestinal barrier		Li L. et al. (2022)
Yinxingye/Ginkgo Folium/Ginkgo Leaf	Ginkgolide B	Increased abundance of *Bacteroides* and decreased abundance of *Helicobacter* and inhibited the mRNA level and protein expression of FMO3, and decreased the concentrations of TMA and TMAO.		Lv et al. (2021)

### 4.1 The mechanism by which traditional Chinese herbs regulate the gut microbiota

Traditional Chinese herbs regulate the diversity of the flora through direct contact with the gut microbiota. Oral administration of herbal tonics is the traditional method of administration in TCM. Interestingly, this creates enough time and space for the herbal ingredients to interact with the gut microbiota (Zhao et al., 2012). It has been found that there are hundreds of components in herbs, such as alkaloids, polysaccharides, glycosides, tannins, and enzymes (Zhu et al., 2021a). Most of these components are not directly absorbed in the gut but become metabolic substrates for intestinal bacteria (Feng et al., 2019a). Similar to prebiotics, these herbal ingredients have the ability to inhibit the multiplication of pathogenic bacteria, promote the growth of probiotics, and regulate microbial metabolites in the gut (Zhang et al., 2012; Zhao et al., 2012; Guo et al., 2015; Huang et al., 2016; Liu et al., 2018; Nie et al., 2019; Habtemariam, 2020; Wang et al., 2022a). Chinese herbs maintain a healthy gut environment by promoting bowel movement and stimulating bowel movements. For example, Xinglou Chengqi Decoction (XCD) and Tanhuo decoction (THD) are both effective in improving gut dysfunction caused by AIS, reducing symptoms such as bad breath, thick tongue, bloating, dry stools or poor bowel movements (Chen et al., 2020; Wang et al., 2021; Guo et al., 2022). The gut motility-promoting effect of Chinese herbs may be related to their stimulation of the release of intestinal neurotransmitters. Anthraquinone glycosides, one of the main active ingredients of the Chinese medicine rhubarb, are barely absorbed into the intestinal tract as a prototype. Anthraquinone glycosides have been found to improve the imbalance of intestinal neurotransmitter release and catabolism caused by disorder of gut microbiota, increase the levels of 5-HT and 5-HIAA in the gut, and thus promote intestinal motility (Guo et al., 2021b).

### 4.2 The significance of TCM in regulating the gut microbiota

By regulating the gut microbiota, Chinese herbs have been found to influence the metabolic levels of intestinal flora metabolites, improve the inflammatory environment in the gut, reduce neuronal apoptosis, and protect the integrity of the intestinal barrier.

#### 4.2.1 Influence on microbial metabolite expression

Reduced metabolic levels of SCFAs, particularly acetate and butyric acid, showed a positive correlation with the severity of AIS (Chen et al., 2019b; Tan et al., 2021). Several studies have found that Chinese herbs can upregulate SCFA levels to improve the prognosis of AIS by correcting disorders of the gut microbiota. THD makes SCFA-producing flora such as *Blautia, Coprococcus, Streptococcus, Dorea, Streptococcus, Bifidobacterium, and Lactobacillus* more accessible to healthy people (Guo et al., 2022). XCD can upregulate *Verrucomicrobia* and *Akkermansia* abundance to increase the levels of enteric butyric acid (Gao et al., 2021). The PLR&CXR combination increases the abundance of *Oscillospira, Lachnospiraceae, and Akkermansia* to increase butyrate release (Chen et al., 2019a). In a study of herbal compounds, researchers found that supplementation with puerarin, astragaloside IV, and berberine (BBR) significantly replenished the intestinal flora producing SCFAs and upregulated the relative levels of acetic acid and butyric acid (Wu et al., 2020b; Wu et al., 2020a; Gong et al., 2021). On the other hand, plasma TMAO concentrations were positively associated with the incidence of adverse clinical outcomes in AIS (Zhai et al., 2019; Tan et al., 2020). BBR was found to downregulate the abundance of Bilophila in the gut and reduce the expression of TMAO-related enzymes to decrease the level of TMAO (Wu et al., 2020a). Enema with an aqueous extract of rhubarb inhibits the abundance of pathogens such as Lachnospiraceae and Romboutsia and reduces serum TMAO and trimethylamine (TMA) levels in rats (Ji et al., 2021). Ginkgolide B (GB) inhibits the mRNA and protein expression of FMO3 and reduces the concentrations of TMA and TMAO (Lv et al., 2021). Based on quantitative analysis of predicted genes, THD was found to reduce the biosynthetic genes of trimethylamine (TMA), a precursor of trimethylamine N-oxide (TMAO), and to enhance trimethylamine-nucleotide protein comethyltransferase (mttB)-related genes, which breakdown TMA into methane (Guo et al., 2021a).

#### 4.2.2 Reducing gut-brain inflammatory injury and neuroapoptosis

The intestinal immune environment has an important role in regulating inflammatory damage to brain tissue after AIS. Increasingly, studies have shown that by regulating gut microbiota, Chinese herbs can effectively improve the intestinal immune environment and reduce brain and intestinal inflammatory responses. At the cellular level, the HQ-HH combination reduces the number of TH17 cells in the gut, increases the number of Treg cells, and reduces inflammation in the gut and brain tissue (Wang et al., 2022b). Tongqiao Huoxue decoction (TQHXD) reduces the number of γδ T cells in the gut and brain, increases the number of Treg cells and attenuates AIS-induced gut-brain inflammatory injury (Zhang et al., 2020). BBR can regulate the Treg/Th17 balance by improving the gut microbiota (Cui et al., 2018). At the molecular level, XCD promotes the release of anti-inflammatory factors such as IL-10 while downregulating proinflammatory factors such as TNF-α, IL-17A and IL-22 (Gao et al., 2021). Qishiwei Zhenzhu pills (QSW) can regulate the relative abundance of Firmicutes and Proteobacteria in the gut, downregulate p38-MAPK and TNF-α and inhibit inflammatory factors such as IL-6 and IL-1β (Fu et al., 2021). Salvia polysaccharides act directly on the gut microbiota to restore the balance between the expression of enteric proinflammatory factors (IL-6 and IL23) and anti-inflammatory factors (IL2, IL10 and TGF-β) (Li et al., 2022a). Lipopolysaccharide (LPS) has been found to promote neuroinflammation in the brain through direct and indirect pathways (Lukiw et al., 2018). THD significantly reduces the number of LPS-producing intestinal flora, such as *Mycobacterium avium*, in the gut, thereby reducing the level of LPS biosynthesis (Guo et al., 2022). It was found that the reduction in AIS-induced neuronal apoptosis by Chinese herbs may be related to the regulation of the gut microbiota. AS-IV reverses the disruption of the gut microbiota caused by AIS and downregulates the expression of the autophagic markers Beclin-1, Atg12, and LC3 II, which is thought to be one of the mechanisms by which AS-IV protects against ischemic brain apoptosis (Xu et al., 2018).

#### 4.2.3 Protecting the integrity of the intestinal barrier

An intact gut barrier is essential to reduce peripheral organ infections caused by the spread of enteric pathogens after AIS (Crapser et al., 2016). Studies have shown that many Chinese herbal medicines, including herbal formulae and compounds, have a reparative effect on intestinal microvillus damage, which enhances the integrity of the intestinal barrier and reduces pathogenic bacterial transfer and endotoxin leakage (Peng et al., 2013; Zhang et al., 2020; Fu et al., 2021; Li et al., 2022a). The protective effect of Chinese herbs on the intestinal barrier is thought to be related to the regulation of key probiotics such as *A. muciniphila* and *Lactobacillus* (Liu et al., 2011; Paone and Cani, 2020). Oral administration of Korean red ginseng (KRG) restores gut microbial diversity and increases the level of *Lactobacillus* (Lee et al., 2022). BBR was found to promote *A. muciniphila* reproduction by stimulating intestinal mucus protein secretion, which forms a dense protective intestinal layer (Dong et al., 2021), and puerarin has been found to have the same effect (Wang et al., 2019). In addition, the tight junctions of intestinal cells are the most important mechanical barriers that maintain the structure, function, and permeability of the gut (Hohman and Osborne, 2022). It was found that Chinese herbs could increase the protein levels of enteric claudins, occludin, and ZO-1 by regulating gut microbiota to contribute to the stability of the intestinal barrier (Chen et al., 2019a; Hu et al., 2022; Wang et al., 2022b; Yan et al., 2022). Bile acids play an important role in maintaining the stability of the gut microbiota and the balance of the immune system of the intestinal mucosa (Sun et al., 2021). It was found that HQ-HH could affect the expression of FXR, a bile acid regulator in the small intestine, by regulating gut microbiota to reduce enteric bile acid levels, thereby reducing bile acid-mediated gut immune injury and hence barrier homeostasis disruption (Goodwin et al., 2000; Wang et al., 2022b).

### 4.3 Gut microbiota helps herbs to be effective

It has been found that the gut microbiota interacts with Chinese herbs and that many herbal compounds need to be transformed into more active and bioavailable molecular structures with the cooperation of gut microbiota (Gong et al., 2020; Qu et al., 2021; Zhang et al., 2022). The rich and numerous microbiota secrete a wide range of enzymes, which may have a significant impact on the stability of the molecular structure of herbal compounds (Wang et al., 2011). For example, anthraquinone aglycones, a poorly absorbed ingredient of rhubarb, can be converted into anthraquinone condensates (anthraquinone aglycones) by the gut microbiota, which will be easily absorbed and exert an anti-ischemic effect (Li et al., 2020a). BBR can be broken down by the gut microbiota into DhBBR and enter the intestinal tissues, where DhBBR will be reduced again to BBR and enter the circulation, and this microbiota-dependent absorption process can significantly improve the bioavailability of BBR (Wang et al. 2022a). Ginsenosides Rb1, Rb2, and Rc can be converted by the gut microbiota to the more biologically active compound K [20-O-beta-D-glucopyranosyl-20(S)-propanediol] (Qi et al., 2011). Fermented ginsenosides were found to have higher anti-inflammatory effects, reducing the LPS-induced increase in the pro-inflammatory cytokines IL-6, TNF-α, and IL-1β and alleviating the intestinal inflammatory response through the TLR4/MAPK signaling pathway (Fan et al., 2021). The malic acid in Pueraria lobata can be fermented to lactic acid by *Bifidobacterium breve*, which may contribute to the enrichment of certain microorganisms with anti-inflammatory properties, such as *Lactococcus* and *Ruminococcus* (Choi et al., 2020). In the presence of gut microbiota, tanshinones and salvianolic acids can undergo metabolic transformations such as methylation, dehydrogenation, and glucuronidation, resulting in higher biological activity and absorption (Cai et al., 2019).

## 5 Discussion

The gut microbiota has an important impact on AIS prognosis, while the microbiota’s significance to gut health should not be overlooked. One study found that depletion of gut microbes by antibiotics alleviated brain injury in MCAO mice; however, the mice that lost their gut microbes invariably developed severe acute colitis after discontinuation of antibiotics (Winek et al., 2016). Traditional Chinese herbs can increase the relative abundance of beneficial flora and decrease the relative abundance of pathogenic flora in the gut, bringing the gut microbiota of AIS patients closer to that of healthy populations. This helps the gut microbiota to play a greater role in positively regulating AIS rather than aggravating it. The study of both Chinese herbal formulae and herbal compounds has its own special significance. For example, herbal compounds can better reveal the mechanisms of interaction between TCM and the gut microbiota, while in the study of herbal formulae, we can see a wider range of synergistic regulatory mechanisms and applications (Ma et al., 2021). Therefore, combining the two seems to be a future direction for continued research into the regulation of gut microbiota by TCM for the treatment of AIS. On the other hand, we found some differences between the available reports, especially for the diversity of gut microbiota, which may be related to differences in host species, feeding environment, and diet. We should consider a potential reason that the dominant microbial species in different segments of the gut are different in the same individual.

Most notably, the study of the regulatory effects of Chinese herbal medicine on the gut microbiota could shed more light on the principles of TCM theory. For example, the same treatment for different diseases is a very important and classical theory in TCM. In short, when different kinds of diseases have the same pattern, they can be grouped together as the same Chinese medicine syndrome and treated with the same therapeutic methods (Zhai et al., 2020). Looking back at stroke, hemorrhagic stroke and ischemic stroke are completely different diseases, but they will be treated in the same way under the guidance of TCM theory (Chang et al., 2016). To investigate the reasons, apart from the currently known antioxidative stress, antiapoptotic, anti-inflammatory, and antiangiogenic effects (Zhu et al., 2021a; Duan et al., 2022), the regulatory effect of Chinese herbs on the gut microbiota may be a potential mechanism. It has been found that both ischemic and hemorrhagic strokes lead to intestinal dysfunction and dysbiosis of the gut microbiota, which can further aggravate neurological injury and induce serious complications (Xu et al., 2021b; Zhang et al., 2021). Traditional Chinese herbs can improve the prognosis of stroke by correcting disorders of the gut microbiota, regulating the expression of microbial metabolites, maintaining intestinal immune homeostasis, and repairing gut barrier damage. Therefore, an in-depth study of the interaction between gut microbiota and Chinese herbs can be a complement to the “same treatment for different diseases” theory of TCM.

In summary, TCM has the advantages of multipathway, multitarget, and synergistic treatment for AIS. Exploring the interaction between Traditional Chinese herbs and gut microbiota is a novel idea that is important for elucidating the mechanism of Traditional Chinese herbs in treating AIS. Meanwhile, this study can help bring the clinical value of TCM into play to a greater extent and has broad research potential.
